# Trend of clinical trials of new drugs for rare diseases in China in recent 10 years

**DOI:** 10.1186/s13023-023-02713-6

**Published:** 2023-05-11

**Authors:** Ai Peng, Xue Fan, Linling Zou, Huan Chen, Jin Xiang

**Affiliations:** 1 Department of Neurology, West China Hospital, Sichuan University, Sichuan, China; 2 Clinical Trial Center, West China Hospital, Sichuan University, Sichuan, China; 3 NMPA Key Laboratory for Clinical Research and Evaluation of Innovative Drug, West China Hospital, Sichuan University, Sichuan, China; 4 Key Laboratory of Drug Targeting and Drug Delivery Systems, West China School of Pharmacy, Sichuan University, Chengdu, 610041 China

**Keywords:** Clinical trials, Rare disease, Development, China

## Abstract

**Background:**

Rare disease is a general term for a disease that affects a small number of people but recognized as a global public health priority. Governments worldwide are paying more and more attention to the academical research and drug investment of rare diseases. The conduct of rare disease clinical trials is still difficult, despite the promotion of government policies and the awakening of social consciousness. In this article, we outlined the characteristics and obstacles of clinical trials of rare diseases in China and expected to provide reference for subsequent clinical trials in this field.

**Results:**

In recent years, China has made some progress in clinical trials of rare diseases in the past 10 years. There were 481 clinical trials on rare diseases in total, covering more than 10 rare diseases with high incidence. Clinical trial applications on rare diseases for a total of 481 were submitted and with an average annual growth rate of 28.2% from 2013 to 2022. The number of clinical trial application for rare diseases in 2016 dramatically increased by 80% compared to 2015 due to the policy document issued by China for clinical research in rare diseases in 2015. Besides, about 70% of applications registering for clinical trials could recruit subjects as expected. Despite this, the number of clinical trials of rare diseases in China was less compared with the United States, Europe and Japan, and the types of infant drugs were limited to biological products and chemical drugs lacking other new treatments.

**Conclusions:**

Efforts have been made in recent years to develop clinical research on rare diseases in China. The number of clinical trials for rare diseases in China was growing steadily every year, which was inseparable from the support of the country, society and rare disease patients. Still, there was a large gap between China and other developed countries in this field and this merit further investigation.

## Introduction

Rare diseases have a large impact on human health worldwide, despite their low prevalence, and the development of treatment for these diseases places a substantial burden on health care budgets [1]. There is no consensus about the definition of rare diseases, and it varies in different regions. In the USA, a rare disease is defined as one that affects no more than 200,000 individuals nationwide and in Europe has a prevalence of up to five per 10,000, or around 250,000 individuals affected. In Japan, rare diseases are those that affects fewer than 50,000 patients. In China, the latest definition of rare disease was released in 2021 at the third multidisciplinary expert seminar on the definition of rare diseases/orphan drugs. Rare disease is defined as diseases with an incidence of less than 1/10,000, a prevalence of less than 1/10,000, and a number of patients less than 140,000 [2–4]. Although individually rare, approximately 6–8% of human population are suffering from rare diseases. Most of rare diseases are chronic and often severely disabling, leaving patients with these diseases with shorten life span, painful life, and hopelessness [5, 6].

Many obstacles and challenges faced in conducting clinical trials, especially for rare diseases. Both commercial and academical clinical trials shared questions around designing of trials, recruiting, applying for regulatory approval and so on. Due to the small population of patients with rare disease and the wide distribution of the population, it is time costing for individuals to attend trials visits. Besides, the complex medication and diaries, recording symptoms or side-effect, adds burden to the patients they already face. Thus, a great number of patients are reluctant to participate in a clinical trial. Furthermore, a meaningful and relevant endpoint is significant for approval on clinical trial and infant drug. Nevertheless, lacking understanding of the biology and natural history of rare diseases is a huge daunting [7].

Some efforts have been taken to promote pharmaceutical industry investment such as regulatory policy to make it easier for drug approval on rare diseases. Over the past decades, more and more governments granted greater priority to rare disease investigation and the development of orphan drugs [8]. In addition, the increased awareness of the community facilitated the development of specialized centers caring for rare disease, patient organizations and rare disease advocacy groups.

Here we summarized the information on rare disease clinical trials in China in the past 10 years, which to some extent reflects the developing trend and obstacles faced by rare disease clinical trials in China. This study aims to provide insights on the development of clinical trials on rare diseases in China and hope to provide reference for the design and development of future rare disease clinical trials.

## Methods

### Source and extraction of data

The data were extracted from the Drug Clinical Trial Registration and Information Disclosure Platform (http://www.chinadrugtrials.org.cn), National Rare Diseases Registry System of China (https://www.nrdrs.org.cn/app/rare/index.html), Chinese Clinical Trial Registry (https://www.chictr.org.cn/), The National Medical Products Administration (https://www.nmpa.gov.cn/), National Medical Products Administration (http://www.nhc.gov.cn/), and Center of Drug Evaluation (NMPA, https://www.cde.org.cn/).

### Data processing and statistical methods

Firstly, the names of rare diseases published in the Chinese First List of Rare Diseases jointly formulated by the National Health Commission and other five departments were retrieved as keywords, and the relevant information on China Food and Drug Administration Registration and Information Disclosure Platform for Drug Clinical Studies from 2013 to 2022 was extracted.

Then data obtained were further grouped following basic information such as the name of the disease, the number of registrations and drugs, research design, trial scope, number of participants, and geographical distribution.

## Results

### Distribution of types of clinical trials for rare diseases

**Fig. 1:**
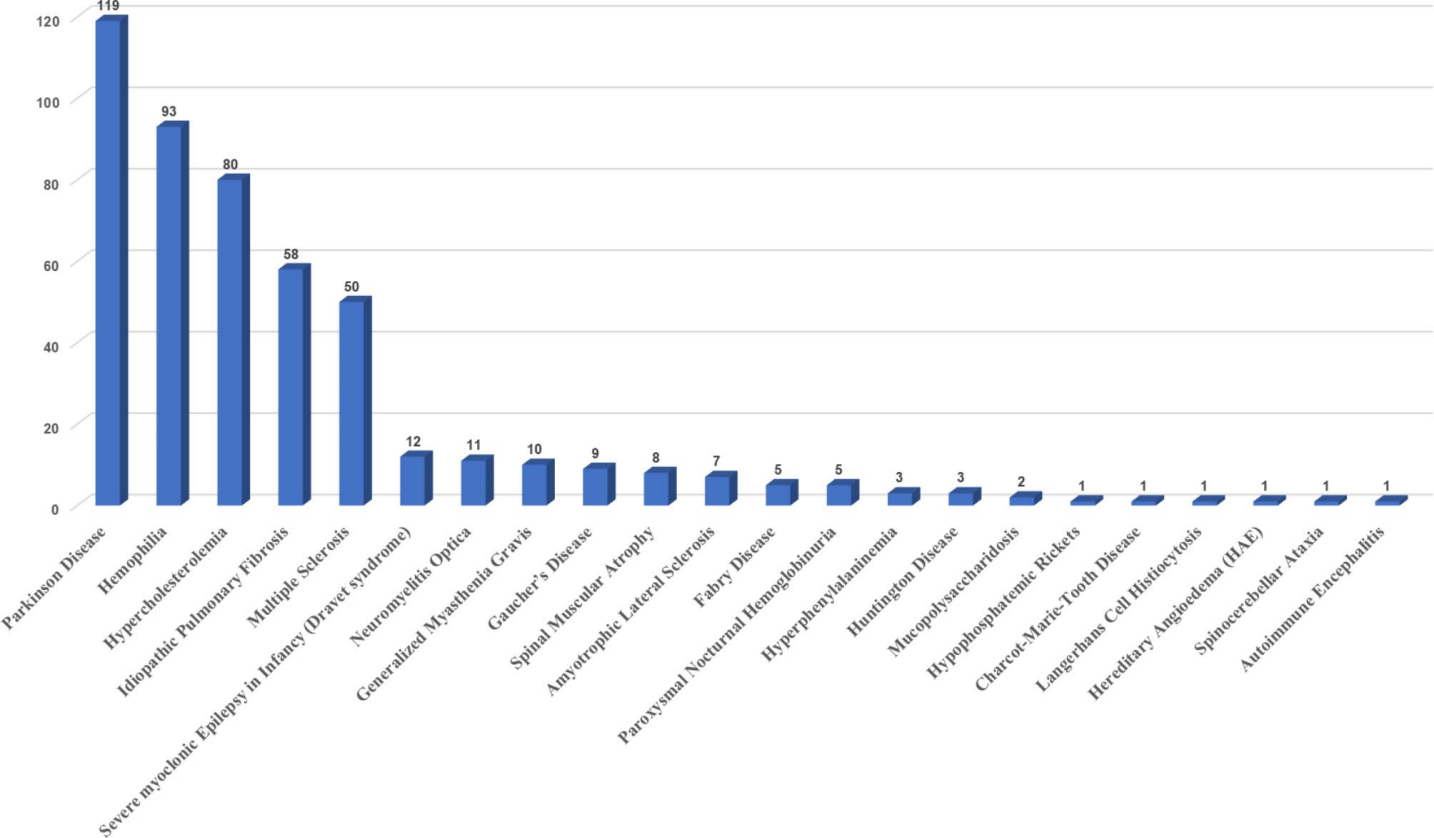
Summary chart of the number of clinical trial applications for each rare disease from 2013 to 2022

As shown in Fig. 1, there were 481 clinical trial applications for rare diseases in China from 2013 to 2022 in total. Among them, the top 5 applications were Parkinson Disease (119, 24.7%), Hemophilia (93, 19.3%), Hypercholesterolemia (80, 16.6%), Idiopathic Pulmonary Fibrosis (58, 12.1%), and Multiple Sclerosis (50, 10.4%), which are categorized in nervous system, blood system, respiratory system, and autoimmune system respectively as shown in Table 1. Rare diseases that are undergoing clinical trials account for 18% (22 species) of the first list of rare diseases (121 species) released in China, declining that most rare diseases in China still lack of clinical research. From the number of trials conducted, we saw Parkinson Disease (PD) was a hot spot in clinical trials in rare diseases. This suggested that a large number of trials were conducted for a relatively small number of rare diseases in China and clinical research may be related to the prevalence of diseases [2].

**Table 1:** Classification of Diseases included in rare disease clinical trials from 2013 to 2022

Category	Disease
Blood system	Hemophilia
	Hypercholesterolemia
Metabolic system	Hypercholesterolemia
	Gaucher’s Disease
	Hyperphenylalaninemia
	Hypophosphatemia Rickets
	Fabry Disease
	Mucopolysaccharidosis
Respiratory system	Idiopathic Pulmonary Fibrosis
Neuroscience	Neuromyelitis Optica
	Severe myoclonic Epilepsy in Infancy (Dravet syndrome)
	Parkinson Disease
	Generalized Myasthenia Gravis
	Spinal Muscular Atrophy
	Amyotrophic Lateral Sclerosis
	Huntington Disease
	Charcot-Marie-Tooth Disease
Autoimmune system	Paroxysmal Nocturnal Hemoglobinuria
	Autoimmune Encephalitis
	Multiple Sclerosis
Hereditary disorders	Charcot-Marie-Tooth Disease
	Hereditary Angioedema (HAE)
	Spinocerebellar Ataxia
Histiocytic diseases	Langerhans Cell Histiocytosis

### Overview of clinical trials for rare diseases

**Fig. 2:**
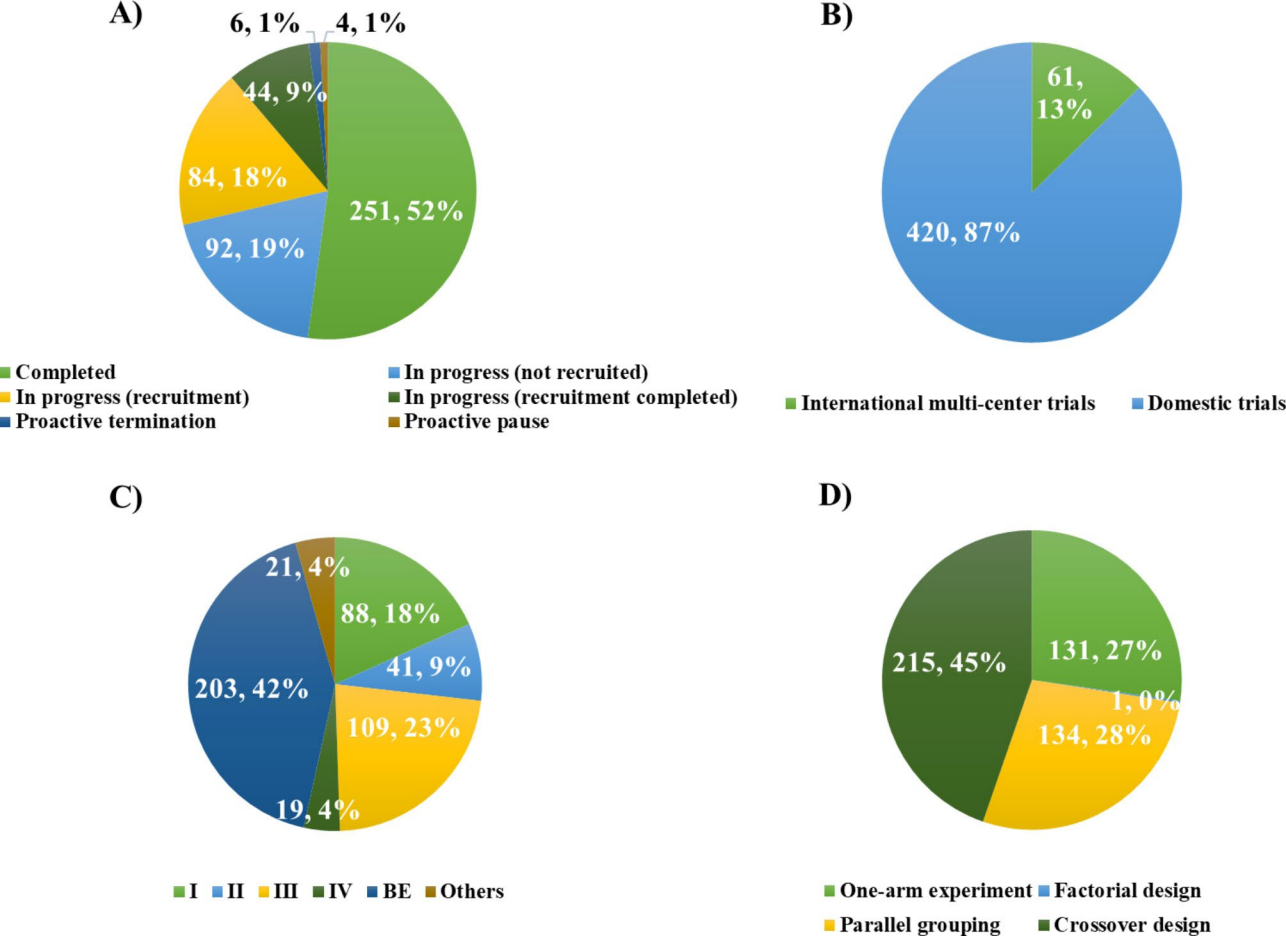
Summary of clinical trial characteristics

A total of 481 clinical trials related to rare disease was registered on the drug clinical trial registration and information disclosure platform, involving 276 enterprises, of which 67 (24.3%) were multinational enterprises. As shown in Fig. 2A), 52% clinical trials have been completed and 46% were in the process of recruiting, 2% were terminated or suspended automatically. Relevant factors leading to this discontinuation of these trials have been concerned, such as safety or toxicity concerns and mostly related to the difficulties in patient enrolment [9]. This reflects the difficulty of recruiting participants with rare disease, resulting in most clinical trials being unable to enter the trial stage as expected.

Most of the clinical trials were still in BE (203, 42%), while phases I, II, III and IV accounting for 18% (88), 9% (41), 23% (109), and 4% (19) respectively as shown in Fig. 2C). From the perspective of clinical trial scope in Fig. 2B), the number of domestic clinical trials was 420 (87%), while the number of international multi-center trials was 61 (13%). Most of the clinical trials in China were bioequivalence trials, indicating that there was a large space for the development of innovative drugs for rare diseases in China.

The trial types and design distributions of clinical trials were shown in the Fig. 2D. In all trials of analyzed, alternative clinical trial designs for studying treatments of rare diseases applied in included single-arm, factorial, parallel grouping and crossover design. In terms of study design, the most popular was crossover design, with a total of 215 studies (45%), and followed by parallel grouping and single-arm experiment, which accounted for 28% (134) and 27% (131) respectively. There was only one case of factorial design, and the proportion was almost non-countable.

**Fig. 3:**
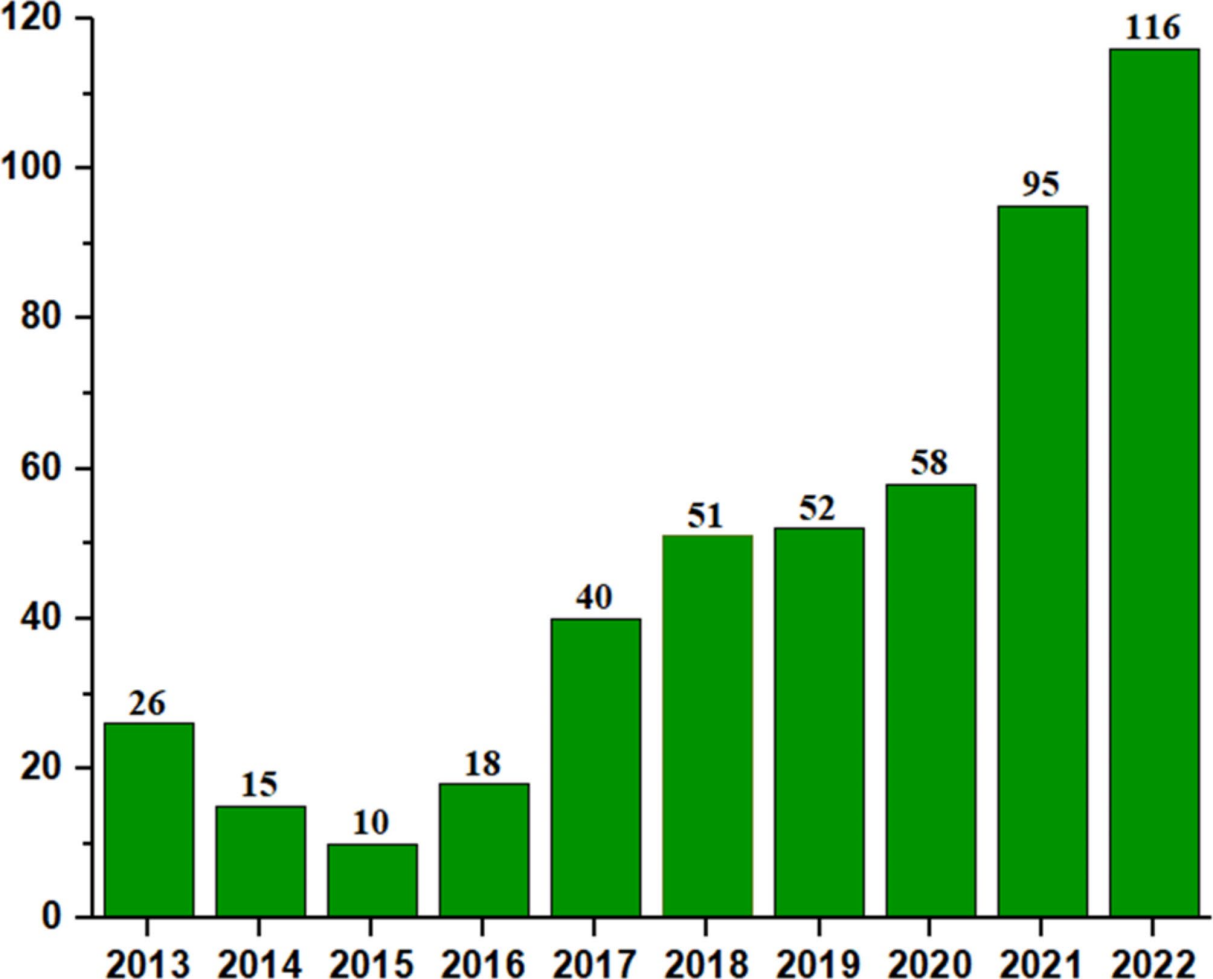
Annual numbers of clinical trial applications for rare diseases in China

Clinical trial applications on rare diseases for a total of 481 were submitted from 2013 to 2022, with an average annual growth rate of 28.2% (Fig. 3). Since 2015, the number of clinical trial registrations for rare diseases has gradually increased. In 2016, the number of clinical trial application for rare diseases increased by 80% compared to 2015, which may be related to the fact that China provided a faster process for clinical trials and infant drug approval for rare diseases in 2015. Notably, the number of applications for rare diseases drugs increased by 122% in 2017 compared with 2016, which was consistent with the overall trend of IND applications [10]. The number of rare disease clinical trial registrations in 2021 increased by 63.8% compared to 2020, while the number of rare disease clinical trial registrations in 2022 increased by 22% compared to 2021. To a certain extent, it could be implied that the outbreak of the global coronavirus epidemic in 2020 has little impact on rare disease clinical trials in China. It cannot be ignored that the annual growth trend of rare disease clinical trials was consistent with the overall development trend of clinical trials in China, but in terms of average annual growth rate, it was still lower than average (32%) [10].

### Characteristics of drugs in clinical trials for rare diseases

**Fig. 4:**
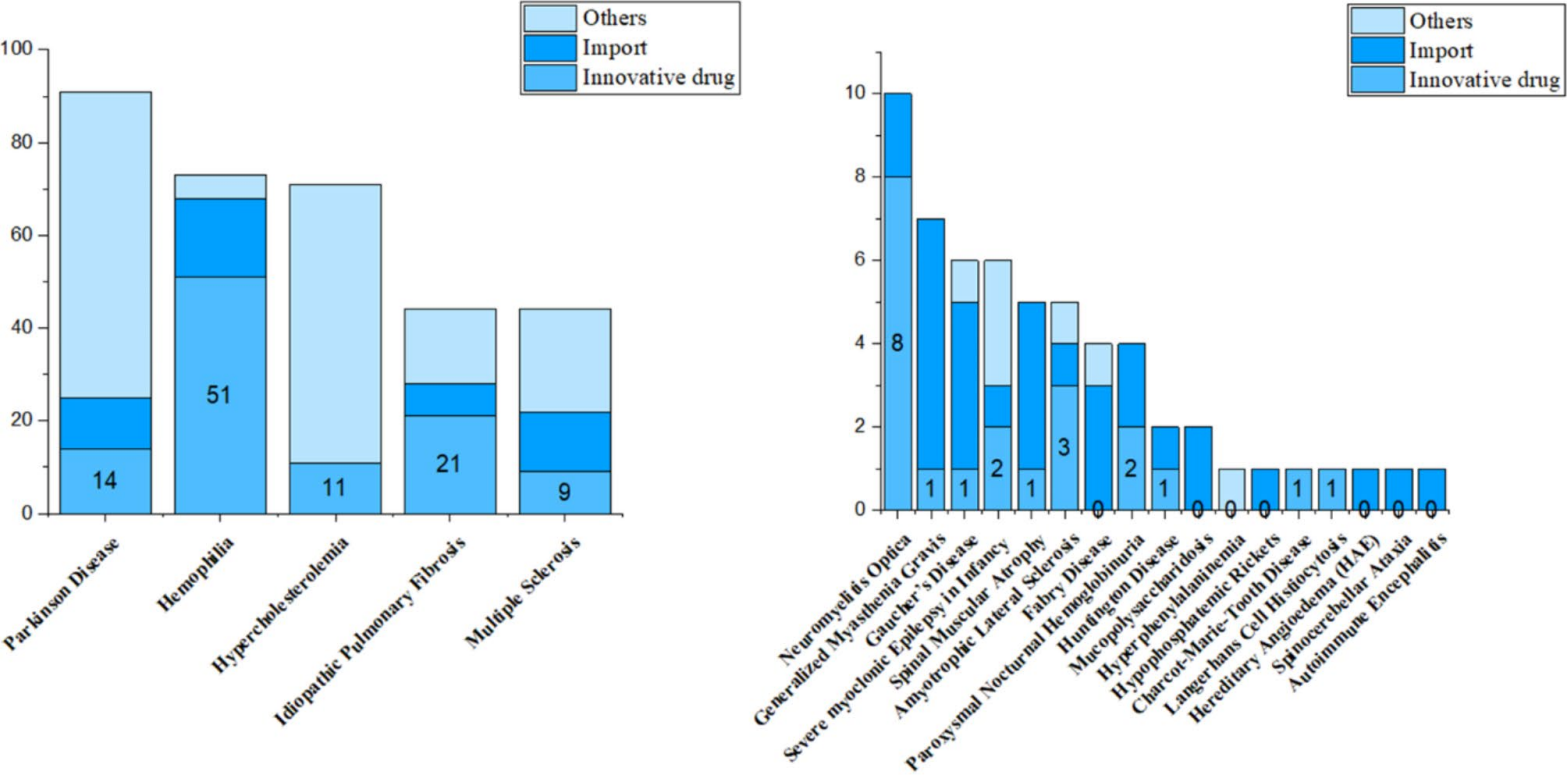
The number of drugs in rare diseases registered in drug clinical trial registration and information disclosure platform

The characteristics of drugs registered were summarized in Fig. 4. The diseases with the most registered drugs were Parkinson disease, Hemophilia, Hypercholesterolemia, Idiopathic Pulmonary Fibrosis, and Multiple Sclerosis, which containing 91, 73, 71, 44, 44 drugs respectively. However, the number of innovative drugs was not corresponding to that of registration. The new drug registered in Parkinson disease, Hemophilia, Hypercholesterolemia, Idiopathic Pulmonary Fibrosis, and Multiple Sclerosis are 14 (15.4%), 51 (69.9%), 11 (15.5%), 21 (47.7%), and 9 (20.5%). Although the largest number of drugs was for Parkinson disease, most were generics and imported drugs. 69.9% drugs were innovative in the registration of Hemophilia. It was assumed that the research and development of innovative drugs for hemophilia by pharmaceutical companies in the recent 10 years was significantly greater than that for other rare diseases. Among the diseases with number of drug registration less than 10, Neuromyelitis Optica owned the highest number of innovative drugs in 8.

**Fig. 5:**
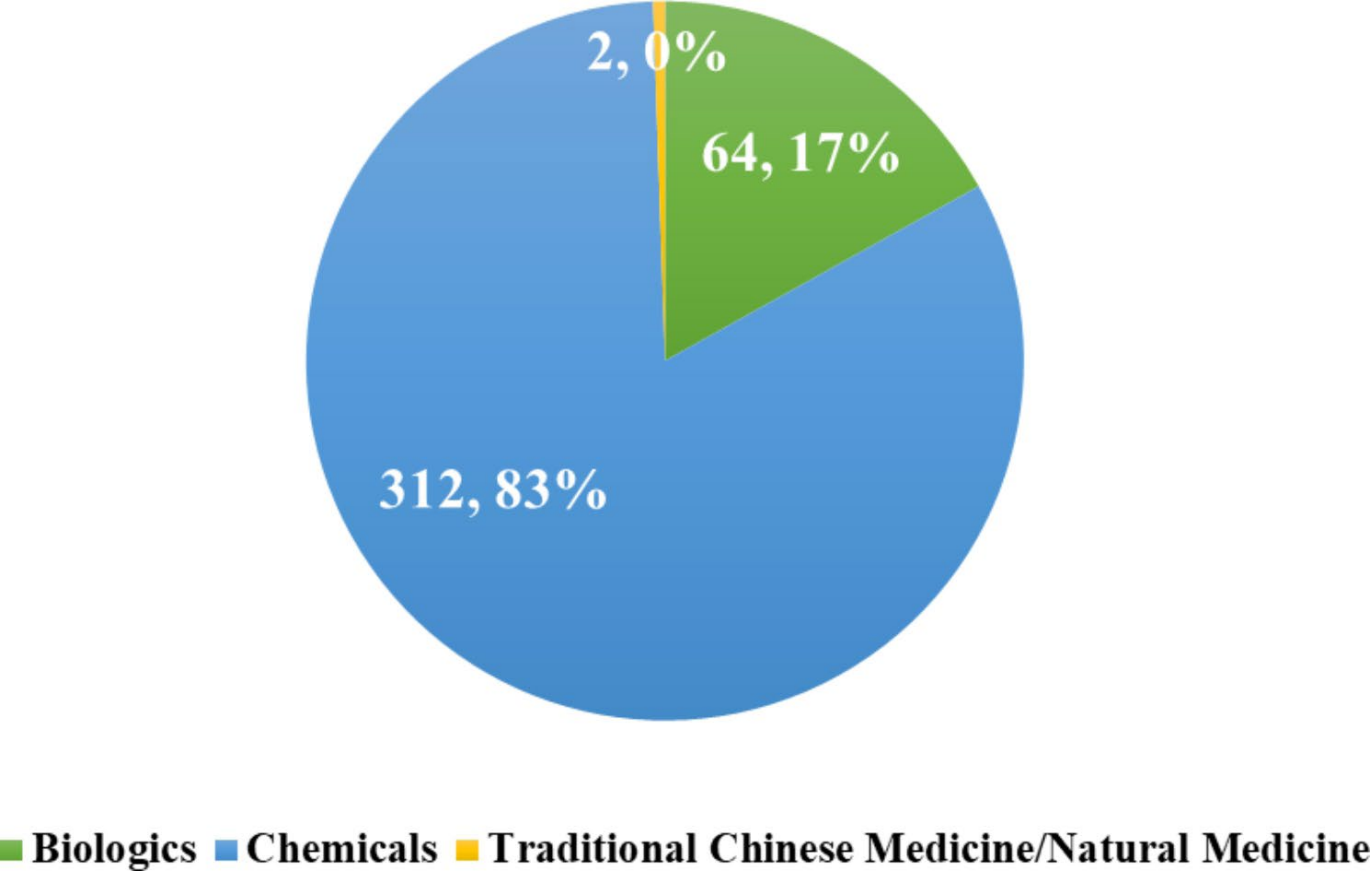
Drug registration classification for clinical trials in rare diseases

In terms of drug types as shown in Fig. 5, of the 481 clinical trials registered, there were 378 drugs in total, and 312 (83%) of which are chemicals and 64 (17%) are biologics. Clinical trials registered in traditional Chinese medicine or natural medicine were the least with only 2 drugs. Drugs for rare diseases were mainly chemical drugs, while biologics accounted for a small part.

**Fig. 6:**
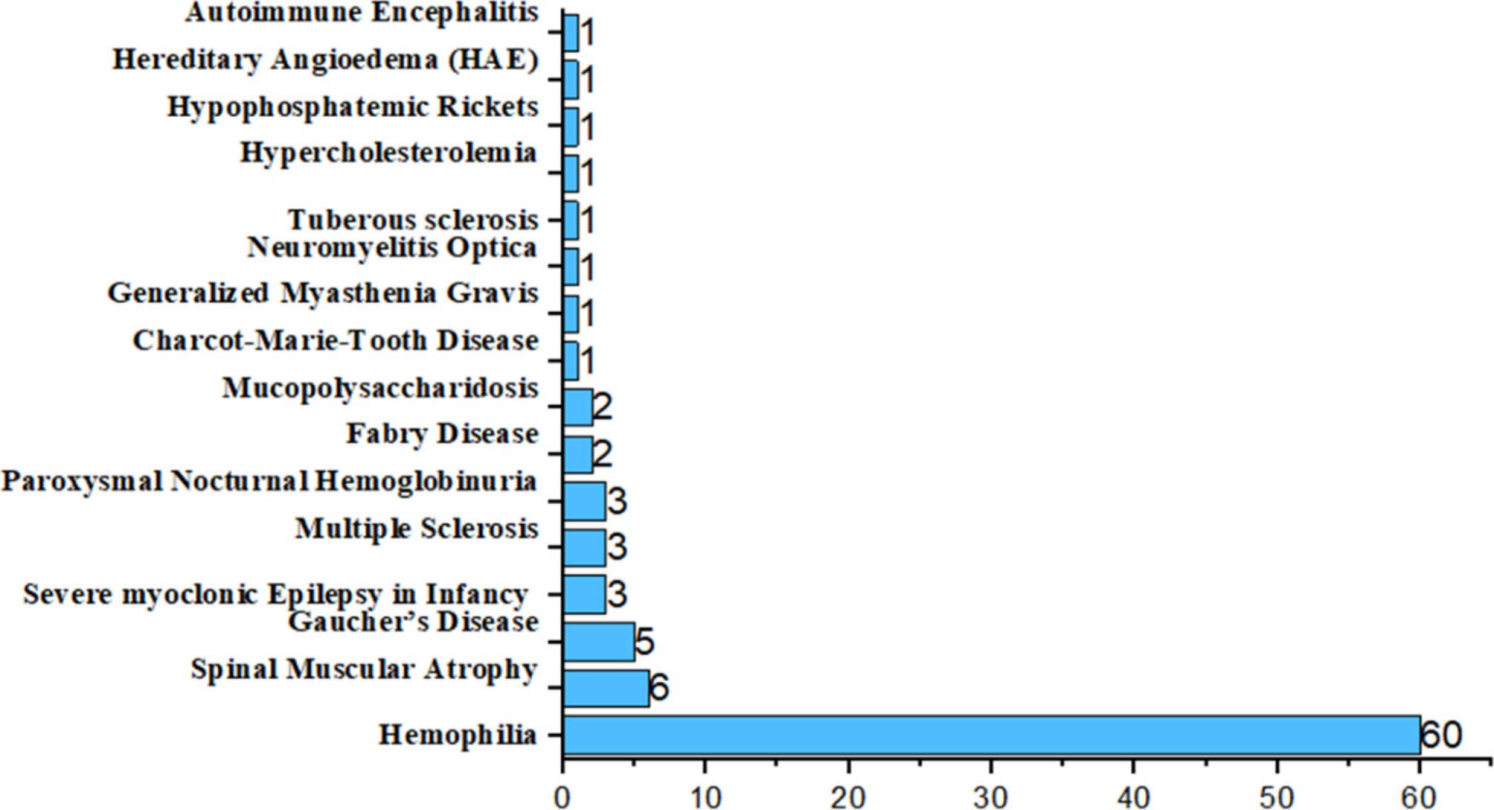
Clinical trials with the age of participants younger than 18 and the corresponding number of drugs in diseases

The clinical trials in which the participants were under 18 years old and were in phases II, III, and IV were statistically analyzed and shown in Fig. 6. The total number of applications was 92 (19% of 481), including dramatically 60 for hemophilia. The clinical trials for rare diseases in adolescents were mainly hemophilia, which was consistent with the age of onset of hemophilia. The overall prevalence of hemophilia in China was 2.73/10,0000, and most of the patients were adolescents. Since more than half these diseases begin in childhood [2], the much more applications on hemophilia showed that the country and pharmaceutical companies attached importance to juvenile diseases.

### The size and geographical distribution of clinical trials

Clinical trials that had completed subject recruitment were chosen out from the available data, and the target and actual numbers were compared. Table 2 shows the data about number of participants related to scope of trials, which told us that domestic clinical trials not only outnumbered international multi-center trials, but also obtained a higher percentage (74.2% vs. 68%) in trials that the actual number of participants was equal to or greater than the target number. The reason why the recruitment of participants in international multi-center trials was not as successful as in domestic clinical trials was speculated to insufficient collaboration between centers related. The ease of recruitment of participants is related to the prevalence of the rare disease and the scattered geographical distribution of the patients also add difficulty to the enrollment of the subjects. In light of the small size of subjects recruited, study designs must be improved to efficiently use the limited samples available.

**Table 2:** The number of participants related to scope of trials

Scope	Number of participants			
	Target>Actual	Target=Actual	Target=Actual	NA
Domestic trials	22	34	35	2
International multi-center trials	6	10	5	1

**NA**: No participant information

**Fig. 7:**
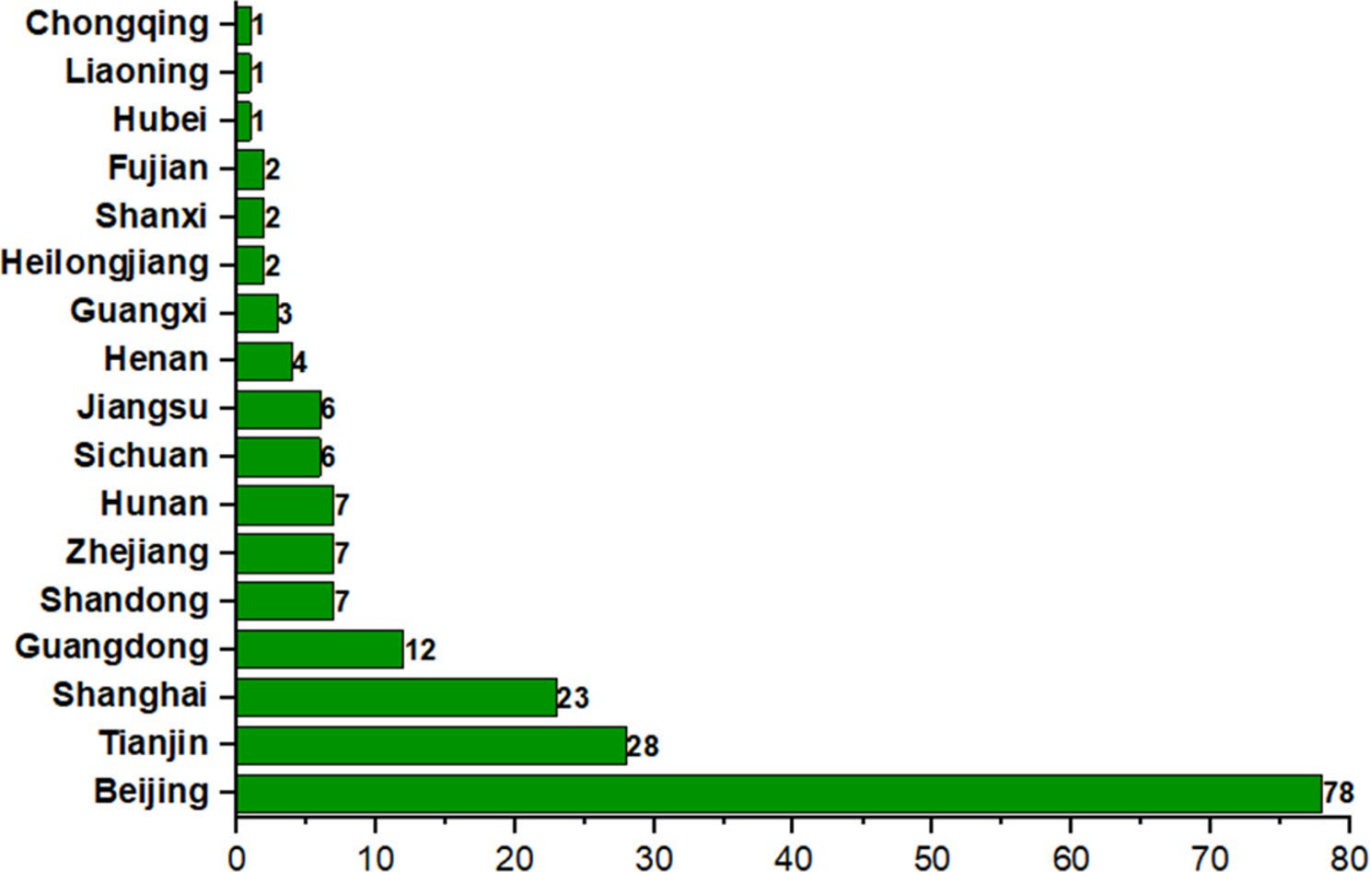
Geographical distribution of leading PI in multicenter clinical trials

**Fig. 8:**
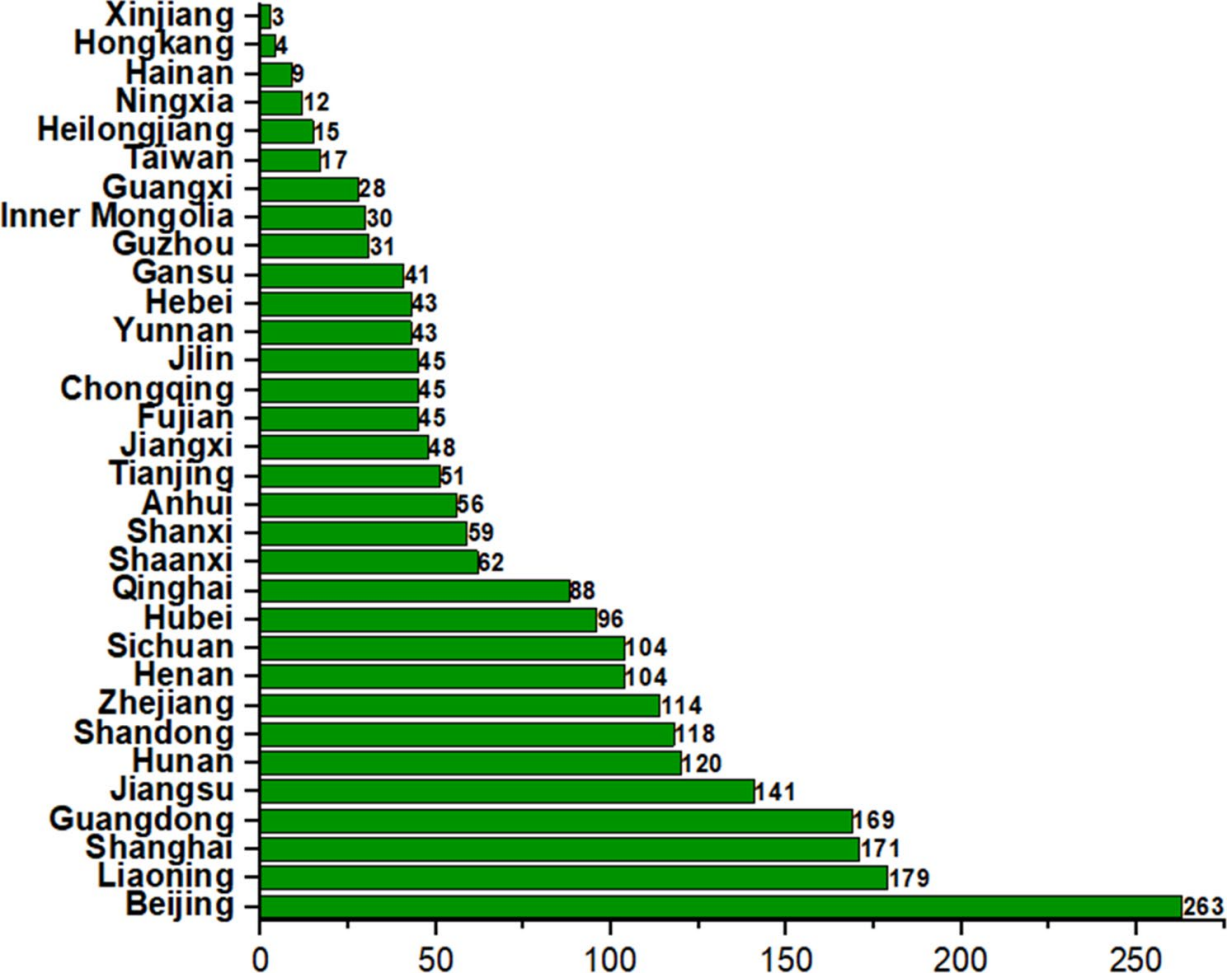
The number of clinical trials for rare diseases in each province in China

In addition, as can be seen from the column of the province where the leading pricinple investigator (PI) of the multi-center clinical trial was located (Fig. 7), most of the leading PIs were from economically developed southeast region of China such as Beijing, Shanghai, Guangdong, and Tianjin. There were 141 (74.2%) leading PIs located in Beijing, Shanghai, Guangdong, and Tianjin. On the one hand, the level of economic development of local regions would affect the development of medical industry, hence to a certain level, the propotion of leading PIs in other provinces in China was smaller. On the other hand, the difficulty of disease treatment and clinical trials conduction of rare diseases is bigger than that of common diseases, which decreased the probability of other provinces to be the leading PIs. The number of rare disease clinical trials conducted in each region shown in Fig. 8. Sites conducting clinical trials of rare diseases were distributed in some coastal cities and economically developed cities, which was consistent with the results of leading PIs. Although there were clinical trials of rare diseases located in western provinces of China, the numbers were dramatically less compared to that in coastal cities, indicating that clinical research on rare diseases still has a lot of space for improvement in these provinces, such as Xinjiang, Ningxia, Inner Mongolia.

## Discussion

This study conducted a statistical analysis of the clinical registration trials of rare disease and drugs registered on the China Food and Drug Administration Registration and Information Disclosure Platform for Drug Clinical Studies from 2013 to November 2022. The results showed that the total number of clinical trials of rare disease and orphan drugs in China, which was less compared with the USA (1252, 1332), Europe (250, 172) and Japan (22, 29) [11], and the research and development of innovative drugs only involved around 10 rare diseases.

The numbers of rare diseases with clinical trials and drugs in test accounted for a small part of the first list of rare disease released in China, and most of them are diseases with high prevalence. The main reason for this phenomenon is that the incidence of most rare diseases is low and there are difficulties in recruiting patients.

In China, overall, the trials and drugs were limited to a small number of rare diseases, and most were stocked in hemophilia. For most of rare diseases, a small proportion of trials was in Phase 3 or Phase 4 (less than 30%) while Phase 3 and Phase 4 trials were considered as promising for drug development, implying there was a huge hurdle to clear on drug development in China.

Of the 481 trials, trials design methods included single-arm, factorial, parallel grouping and crossover design. 45% were trials of crossover design and 27% were single-arm trial and 28% were parallel grouping. The essential feature between a crossover trial and a conventional parallel-group trial is that each patient serves as his/her own control. In crossover trials, each participant would receive all compared treatments in a random order. The effects of two or more treatments can be observed in the same individual, so it can effectively control irrelevant variables and thus reduces the sample size needed [12]. Thus, due to the limitation of the number of subjects, the crossover design was applied in most rare disease clinical trials.

Drugs for rare diseases in trials are mainly chemical drugs (312, 83%), followed by biological drugs (64, 17%). The majority of biological drugs are proteins, lacking nucleic acid preparations, gene and cell therapies and vaccines. Due to the complex pathological process, the treatment of rare diseases should be diversified, rather than limited to small molecule chemical drugs or some simple protein preparations. China is known worldwide for its distinctive TCM theories [13, 14]. In order to diversify the treatment of rare diseases and improve the cure rate, the Chinese government, research institutions and pharmaceutical companies need to pay attention to the advantages of traditional Chinese medicine and combine it with clinical research on rare diseases.

For most rare diseases, imported drugs accounted for a considerable proportion, thus, drug-accessibility and affordability might cause barries for domestic patients to use them. It suggested that the needed drugs for rare diseases in China market is far greater than the pharmaceutical market could provide, and therefore it is necessary to develop clinical research on rare diseases vigorously .

To fuel the clinical research on rare diseases, China has issued a series of policies for drug research and development and clinical research for rare diseases. One of the policies was to prioritize and accelerate the review and approval of rare disease-related registrations. For example, in 2015, the State Food and Drug Administration issued the “Announcement on Several Policies for Drug Registration, Review and Approval”, clarifying that the registration application of innovative drugs for rare diseases can be accelerated on the process of review and approval. In 2017, “Opinions on Deepening the Reform of the Review and Approval System and Encouraging Innovation in Drugs and Medical Devices” put forward clear opinions on encouraging the research and development of drugs for rare diseases and accelerating the registration and approval of rare disease drugs. In 2018, China listed rare disease treatment drugs as data protection objects and granted a 6-year data protection period from the date when the indication was first approved in China. In addition, to improve the level of diagnosis and treatment of rare diseases and safeguard the health rights of patients with rare diseases, China has established a national cooperation network for the diagnosis and treatment of rare diseases. In 2020, the Preferential Review and Approval Procedures for Drug Marketing Authorization issued by the National Medical Products Administration pointed out that the review time limit for general drug marketing authorization applications is 200 days, and the review time limit for drug marketing authorization applications included in the priority review and approval procedures is 130 days. The evaluation time limit for rare disease drugs that have been marketed abroad and have not been marketed domestically is 70 days. As China and the society paying more and more attention to rare diseases, at the Fourth Session of the 13th National People’s Congress, it was officially stated that China would further improve the diagnosis, treatment, and medical security of rare diseases, increase support for the research and development of innovative drugs and generic drugs for rare diseases, and strive to improve the dilemma of domestic rare disease patients drug using difficulties. In the contrary, The Orphan Drug Act of 1983 was passed by US Congress to give financial incentives to pharmaceutical companies including market exclusivity and tax breaks to develop drugs for rare diseases, which is 30 years earlier than China [4]. The drug investigation on rare diseases in the US, EU and Japan was much more promising and vigorous [11].

At present, there is still a certain gap between domestic and foreign countries in drug research and development for rare diseases. Through the data analysis of this study, most of the domestic clinical research on rare disease drugs was bioequivalence tests and the drugs about rare diseases in China were non-innovative drugs. Conversely, one study showed that 48% of clinical trials registered on Clinical Trials. gov about rare diseases were in the United States. The disparity in rare disease research at home and abroad is mainly due to the late start of biomedical research in China, the low level of basic research, the weak ability of independent innovation, and the backwardness of scientific and technology, so the research and development of innovative drugs for rare diseases is also lagging. Nevertheless, in light of the annual growth rate (28.2%) of the number of clinical trial applications for rare diseases, it was apparent that the Chinese government and society were paying more and more attention to clinical research on rare diseases, and the number of clinical trials was showing a steady growth trend year by year.

Based on various policies promulgated by the country, the research environment for rare diseases has greatly improved in recent years. During the period of 2018–2022, the National Medical Products Administration has successively issued relevant policies such as the Technical Guiding Principles for the Acceptance of Data from Overseas Clinical Trials of Drugs, the Drug Administration Law of the People’s Republic of China, and the Statistical Guiding Principles for Clinical Research of Drugs for Rare Diseases (Trial Implementation). These policies clearly state that: for drugs that are used for rare diseases and lack effective treatment methods, the overseas clinical trial data can be conditionally accepted after evaluation. At the same time, it also encourages pharmaceutical enterprises to develop novel drugs for rare diseases and promote the progress of drug technology. For the clinical research of drugs for rare diseases, it may be considered to relax the inclusion and exclusion criteria to allow relatively more patients to enter the study. However, due to the complexity of rare diseases, small number of rare disease patients, the difficulty of recruitment, and the high cost of treatment, there are still many obstacles to the clinical research of rare diseases [4]. To boost more improvements of infant drug clinical trials on rare diseases, it is not enough to depend on the government separately. The development of specialized centers caring for rare disease patients within academic medical institutions and partnerships between academic institutions, health care providers and the pharmaceutical industry should also pay attention to it either [8].

This retrospective study still has some limitations. The database is limited and does not include the registration information from other platforms, so it can only reflect the clinical trial of rare diseases research status of the drug clinical trial registration and information disclosure platform. This study only reflects the registered clinical trials from 2013 to 2022, it cannot represent the overall level of rare disease clinical research in the future in China. By integrating the information of clinical trials and drugs on rare diseases in China, this work is expected to boost the drug development in rare diseases and offer the latest research trend in China.

## Conclusions

This work described in detail the recent situation of rare disease clinical research in China, analyzed the reasons for its relative lag and important areas for future development. Although China has released many policy documents to provide a rigorous guarantee for the research and treatment of rare diseases, clinical trials of rare diseases are hard to conduct, and the recruitment of participants is still difficult, so clinical research on rare diseases will still be a key field supported by the country in the future. Besides, the development of clinical research on rare diseases is limited by their nature. Due to the complexity of the disease, the study of the definition and standardization of rare diseases requires more investment of time and money and the cooperation of international organizations [11, 15, 16]. In addition, the proportion of traditional Chinese medicine in drugs for rare diseases is very small, and the innovation of rare disease treatment research focusing on traditional Chinese medicine should be increased to promote the diverse development of rare disease treatment. Thus, there is still a long way for China to go in clinical studies in rare diseases and this merit further investigation.

## Data Availability

The datasets analyzed during the current study are available from the corresponding author on reasonable request.

## References

[1] Dawkins HJS (2018). Progress in Rare Diseases Research 2010–2016: an IRDiRC perspective. Clin Transl Sci.

[2] Chang X (2022). A survey of registered pharmacological clinical trials on rare neurological diseases in children in 2010–2020. Front Pediatr.

[3] Burton A (2021). Drug Discovery and Development in Rare Diseases: taking a closer look at the Tafamidis Story. Drug Des Devel Ther.

[4] Mellerio JE (2022). The challenges of clinical trials in rare diseases. Br J Dermatol.

[5] Ferreira CR (2019). The burden of rare diseases. Am J Med Genet A.

[6] Stoller JK (2018). The challenge of Rare Diseases. Chest.

[7] Tambuyzer E (2020). Therapies for rare diseases: therapeutic modalities, progress and challenges ahead. Nat Rev Drug Discovery.

[8] Rudebeck M (2021). Clinical development innovation in rare diseases: lessons learned and best practices from the DevelopAKUre consortium. Orphanet J Rare Dis.

[9] Musters A, Tas SW (2020). Room for improvement in clinical trials for rare diseases. Nat Rev Rheumatol.

[10] Su X (2022). Trends in innovative drug development in China. Nat Rev Drug Discov.

[11] Sakate R (2018). Trends of clinical trials for Drug Development in Rare Diseases. Curr Clin Pharmacol.

[12] Wellek S, Blettner M. On the proper use of the crossover design in clinical trials: part 18 of a series on evaluation of scientific publications. Dtsch Arztebl Int. 2012 Apr;109(15):276–81. 10.3238/arztebl.2012.0276 PMC3345345 22567063

[13] Tang JL, Liu BY, Ma KW (2008). Traditional chinese medicine. Lancet.

[14] Xu HY (2019). ETCM: an encyclopaedia of traditional chinese medicine. Nucleic Acids Res.

[15] Blumenrath SH (2020). Tackling rare diseases: clinical trials on chips. Exp Biol Med (Maywood).

[16] Austin CP (2018). Future of Rare Diseases Research 2017–2027: an IRDiRC perspective. Clin Transl Sci.

